# Sports despite masks: no negative effects of FFP2 face masks on cardiopulmonary exercise capacity in children

**DOI:** 10.1007/s00431-023-05316-2

**Published:** 2023-11-11

**Authors:** Annika Weigelt, Isabelle Schöffl, Kathrin Rottermann, Wolfgang Wällisch, Sarina Katrin Müller, Sven Dittrich, Matthias Jens Hübner

**Affiliations:** 1 Department of Pediatric Cardiology, Friedrich-Alexander-Universität Erlangen-Nürnberg, Loschgestrasse 15, 91054 Erlangen, Germany; 2 School of Clinical and Applied Sciences, Leeds Beckett University, LS13HE Leeds, UK; 3 Department of Otholaryngology, Head and Neck Surgery, Friedrich-Alexander-Universität Erlangen-Nürnberg, Waldstraße 1, 91054 Erlangen, Germany

**Keywords:** VO_2_peak, End-tidal CO_2_, End-tidal O_2_, Oxygen saturation, Treadmill testing, COVID-19, Coronavirus

## Abstract

Face masks were recognized as one of the most effective ways to prevent the spread of the COVID-19 virus in adults. These benefits were extended to children and adolescents. However, the fear of negative consequences from wearing a face mask during physical exercise led to cancellations of physical education lessons. This further decreased the amount of physical activity available to children and adolescents during the pandemic. However, there is little published data on the potential adverse effects of wearing the most effective and partially mandatory FFP2/N95 face masks during PE or physical activity (PA) in this age. Even though the pandemic has been declared as passed by the WHO, the rise of a new pandemic and thus the use of face masks for limiting its spread is inevitable, so we need to be better prepared for alternative options to lockdown and limitation of PA in such a scenario. Twenty healthy children aged 8–10 years performed two identical cardiopulmonary exercise tests as an incremental step test on a treadmill within an interval of 2 weeks, one time without wearing a protective mask and one time wearing an FFP2 mask. The cardiopulmonary exercise parameter and especially the end-expiratory gas exchange for oxygen and carbon dioxide (petO_2_ and petCO_2_) were documented for each step, at rest and 1 min after reaching physical exhaustion. Twelve boys (mean age 8.5 ± 1.4 years) and 8 girls (mean age 8.8 ± 1.4 years) showed no adverse events until maximal exertion. The mean parameters measured at peak exercise did not differ significantly between both examinations (mean peak VO_2_ = 42.7 ± 9.5 vs 47.8 ± 12.9 ml/min/kg, *p* = 0.097, mean O_2_pulse 7.84 ± 1.9 ml/min vs. 6.89 ± 1.8, *p* = 0.064, mean VE/VCO_2_slope 33.4 ± 5.9 vs. 34.0 ± 5.3, *p* = 0.689). The only significant difference was the respiratory exchange rate (RER, 1.01 ± 0.08 vs 0.95 ± 0.08, *p* = 0.004). The measured respiratory gases (end-tidal O_2_ and CO_2_) decreased and respectively increased significantly in almost every step when wearing an FFP2 mask. However, these levels were well below hypercapnia and above hypoxia.

*Conclusion*: In this study, no significant differences in the cardiorespiratory function at peak exercise could be discerned when wearing an FFP2/N95 face mask. While the end-tidal values for CO_2_ increased significantly and the end-tidal values for O_2_ decreased significantly, these values did never reach levels of hypercapnia or hypoxia. Furthermore, the children terminated the exercise at a lower RER and heart rate (HR) suggesting a subconscious awareness of the higher strain. Since the detrimental effects of limiting sports during the pandemic are well documented, stopping PE lessons altogether because of the minor physiological effects of wearing these masks instead of simply stopping pushing children to perform at their best seems premature and should be reconsidered in the future.

**Table:** 

**What is Known:** *• Wearing a face mask has an influence on psychological, social, and physiological functions in adults.* *• Because of the observed effects of wearing face masks in adults, physical activity in children was limited during the pandemic.*	
**What is New:** *• Wearing an FFP2/N95 mask during physical activity did not lead to hypercapnia or hypoxia in children in this study.* *• Even though end-tidal CO2 values were significantly higher and end-tidal O2 values significantly lower when wearing an FFP2/N95 face mask, no pathological values were reached.*

**What is Known:**

*• Wearing a face mask has an influence on psychological, social, and physiological functions in adults.*

*• Because of the observed effects of wearing face masks in adults, physical activity in children was limited during the pandemic.*

**What is New:**

*• Wearing an FFP2/N95 mask during physical activity did not lead to hypercapnia or hypoxia in children in this study.*

*• Even though end-tidal CO2 values were significantly higher and end-tidal O2 values significantly lower when wearing an FFP2/N95 face mask, no pathological values were reached.*

## Introduction

The COVID-19 pandemic was caused by the mutated coronavirus, called SARS-CoV-2, which is transmitted largely by the respiratory route [1]. As a consequence, large portions of the world were affected not only by the clinical consequences but also by social aspects like lockdowns [2, 3] and personal protection through face masks [4–8]. Especially, children suffered physically, socially, and psychologically from the imposed lockdown measures [2; 9–11]. Already early on face masks were recognized as one of the most effective ways to prevent the spread of the virus [5, 12, 13], and the FFP2/N95 face mask proved to be more effective than “normal” surgical face masks [14].

Although limiting the spread of the virus was the most important objective and the use of face masks was therefore essential, there were also several disadvantages that became apparent: (1) in Europe, the previously unusual wearing of face masks led to subjective symptoms like headaches, stress, and discomfort [15]; (2) an increase in pulmonary resistance could be observed [13]; and (3) some participants experienced an increase in dyspnea because of the effect of CO_2_ when rebreathing a small volume of exhaled gas while wearing a face mask [13].

As a consequence, a number of studies focused on the side effects of wearing face masks in adults [4, 6–8, 13, 15–25]. The use of simple cloth face masks led to an increase in dyspnea, but depending on the study the oxygen saturation decreased or stayed idem [25, 26]. The use of FFP2/N95 face masks led to an increase in self-perceived dyspnea [17, 27–29], performance [17, 27], peak oxygen consumption ($\dot{V}{O}_{2}peak$) [30], heart rate (HR) [27], peak minute ventilation ($\dot{V}Epeak$) [30], and oxygen pulse (O_2_pulse) [30] and a decrease in oxygen saturation (SpO_2_) [31]. At the same time, several studies were able to show that the effect of face masks on low- to moderate-intensity exercise was little to negligible [32, 33].

Because of the benefits of wearing face masks in the containment of the pandemic, the use was extended to children and adolescents. After recognizing the side effects of wearing face masks especially during physical activity, many schools limited physical education (PE) lessons or cancelled them altogether. This further decreased the amount of physical activity available to these age groups during the pandemic [2, 9–11, 34]. However, there is little published data on the potential adverse effects of wearing face masks during PE or physical activity (PA) in children and adolescents. So far, no effects on SpO_2_ or retention of carbon dioxide (CO_2_) could be observed during treadmill running [35]. Nor were there any differences in lung function tests or perceived exertion during square-wave tests [36] or sit-to-stand tests [37].

Still, all these studies were limited to cloth face masks and none were conducted using FFP2/N95 face masks which became mandatory in many countries during the pandemic. Furthermore, none of the studies conducted true cardiopulmonary exercise testing (CPET) with objective ventilatory parameters like end-tidal CO_2_ or O_2_ pressures (petCO_2_ and petO_2_) for evaluating the effects of wearing face masks on cardiopulmonary function in children.

## Material and methods

The study was approved by the Ethics Committee of the University of Erlangen-Nuremberg, FRG (480_20B). All study participants as well as their legal guardians gave written informed consent according to the standards set by the Declaration of Helsinki.

### Participants

Children between the ages of 8 to 10 years were enrolled. The children were recruited from local elementary schools and through our website from April to July 2021.

Inclusion criteria were:

- Age between 8 and 10 years
- No underlying chronic disease
- Willing to participate in the study
- Ability to perform a treadmill exercise test
- No acute or chronic infectious disease
- No symptoms of post-COVID-19

Height and weight were measured using a stadiometer and electronic scale (Seca 704 S, Hamburg, Germany); BMI was then calculated using the height and weight of the children. *Z*-scores were calculated according to Kromeyer et al. [38].

### Cardiopulmonary exercise test

Heart rate was recorded using a 12-lead-ECG (Custo^®^) and expired gases were collected breath-by-breath (Metalyzer, Cortex, Germany). Cardiac, ventilatory, and metabolic parameters were recorded and analyzed in the Metasoft Studio (Cortex, Germany).

The exercise test consisted of an incremental age-adapted test protocol on a treadmill (COSMED T 170, COSMED, Italy) [39]. This protocol consists of steps with a length of 2 min. After a rest phase, the starting speed is 3 km/h, which increases to 6 km/h in the second step, 8 km/h in the third step, and then increases further by 1 km/h for every further step. The inclination is at 1% for the simulation of a natural environment. In order to achieve peak exertion, all children were encouraged verbally to run until subjective exhaustion, and all tests were performed by the same researchers.

### Randomization and mask fitting

**Fig. 1:**
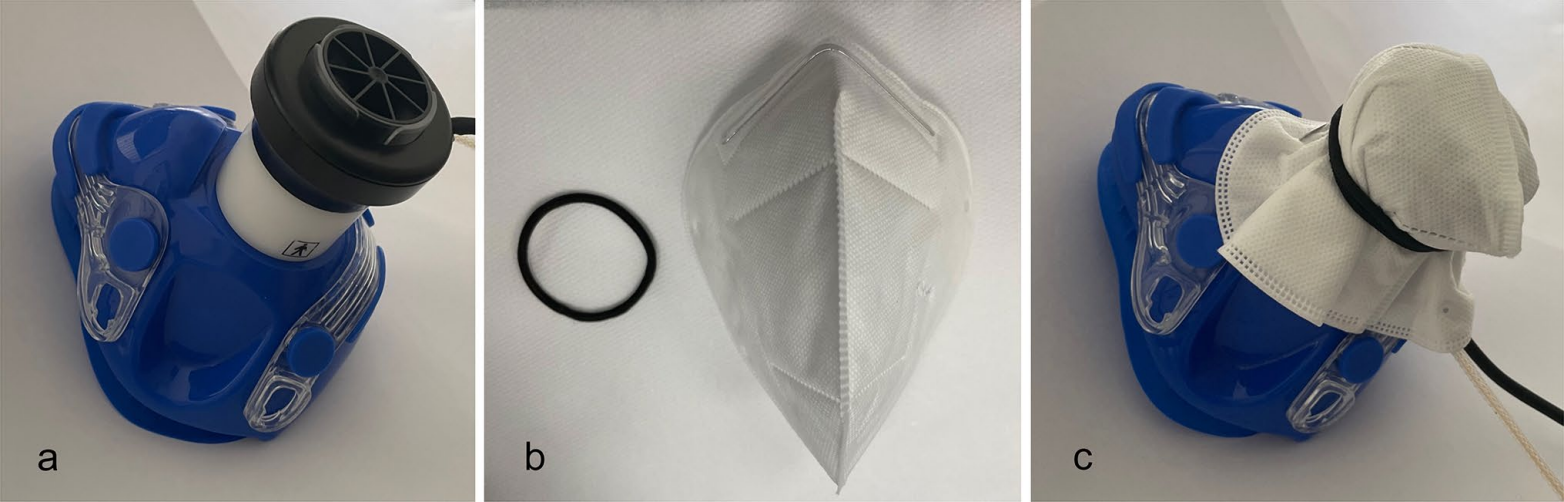
**a** Small low-dead-space respiratory valve (88ml) with a size-matched mouthpiece. **b** FFP2/N95 mask and elastic band. **c** FFP2/N95 mask was fitted over the respiratory valve using the elastic band

The sequence for the two tests was chosen randomly for each child. One test was conducted using the standard mouthpiece with the small respiratory valve like during normal CPET (s. Fig. 1a). For the other test, an FFP2/N95 mask was fitted over the respiratory valve using an elastic band (s. Fig. 1b-c). Securing the mask on the outside of the respiratory valve allowed us to measure the O_2_ and CO_2_ partial pressures as well as minute ventilation on the inside of the FFP2/N95 mask as in a normal condition, since the sample line was attached to the respiratory valve (s. Fig. 1c). The advantage of this design is that it represents the true gas exchange on the inside of an FFP2/N95 mask, but a double-blinded design is impossible.

After completing both tests, each child was asked which test had felt harder. We only used this question to keep subjective feelings simple.

### Measurement of gas exchange

All participants underwent two consecutive cardiopulmonary exercise testings performed at least one but not more than 2 weeks apart. A small low-dead-space respiratory valve (88ml) with a size-matched mouthpiece and headgear was fitted for each child. During the tests, the gas exchange was measured continuously using a breath-by-breath method and averaged over 15-s intervals (Metalyzer 3B, Cortex, Leipzig, Germany). The criteria for completion of a valid peak exercise test were (1) peak heart rate (peak HR) within 5% of the age-predicted maximum, (2) respiratory exchange ratio (RER) ≥ 1.0, and (3) volitional fatigue [40, 41]. We chose a threshold of 1.0 RER for completion of a valid $\dot{V}{O}_{2}peak$ since it is more difficult to achieve higher RER values when testing children [42].

The V-slope method proposed by Beaver et al. [43] was used to determine the ventilatory threshold VT_1_. By plotting $\mathrm{oxygen uptake} (\dot{V}{O}_{2}$) (ml/min) against the logarithm of minute ventilation ($\dot{{V}_{E}}$) (ml/min), the slope of this linear relation through single regression analysis was calculated [40] for determining the oxygen uptake efficiency slope (OUES).

By plotting $\dot{{V}_{E}}$ against carbon dioxide production (${\dot{V}}_{CO2}$) up to the first ventilatory threshold (VT1), the slope (${\dot{V}}_{E}/{\dot{V}}_{CO2}$) was obtained from the regression line [44]. The OUES was also obtained up to VT1.

The breathing reserve was calculated from the FEV_1_ × 35, which approximates the maximal voluntary ventilation (MVV).

PetO_2_ and petCO_2_ were documented for each step, at rest and 1 min after reaching physical exhaustion. These values correspond to the last gas in expiration and can qualify as alveolar gas [45] permitting a direct comparison between the respiratory gas during each step.

### Statistical analysis

Data were collected with Microsoft Excel 2000^®^ and statistical analysis was performed using SPSS 12.0^®^ (SPSS Inc., Chicago, IL). All continuous variables are reported as means and standard deviations when they were normally distributed, otherwise as median and interquartile range. All categorical data are reported as absolute numbers and in percent of the group. The Kolmogorov–Smirnov test was used to check for normal distribution. The homogeneity of variance was investigated using Levene’s *F*-test. Normally distributed variable differences gained with and without an FFP2 mask were assessed with paired *t*-tests; otherwise, the Wilcoxon or the Whitney–Mann *U* tests were used. For the comparison of the petO_2_ and petCO_2_ values with and without mask, Bland–Altman plots and Lin’s coefficient were used. Missing values were not included in the analyses. Due to the lack of clinical trials in this area, the comparative changes of parameters in such settings are unknown. Therefore, no sample size calculation was performed. This study will also serve as a basis for power calculations for future trials.

Statistical significance was set at *p* < 0.05.

## Results

### Participants

We tested 20 healthy children without chronic or recent illnesses (8 girls and 12 boys). The anthropometric data are illustrated in Table 1.

**Table 1:** Anthropometric data as well as extracurricular sports participation as means and standard deviation (SI units in brackets). *Z*-scores are calculated according to Kromeyer et al. [38]

	**Girls**	**Boys**
*n*	8	12
Age (years)	8.5 ± 1.4	8.8 ± 1.4
Height (cm)	140.1 ± 8.4	135.3 ± 7.3
Height (*z*-score)	42.2 ± 21.1	78.8 ± 14.8
Weight (kg)	33.1 ± 5.8	28.7 ± 3.9
Weight (*z*-score)	35.3 ± 23.9	66.1 ± 20.1
Body mass index (kg/m^2)^	16.7 ± 1.6	15.6 ± 1.2
Body mass index (*z*-score)	52.2 ± 24.6	33.3 ± 23.8

### Cardiopulmonary exercise test

All data from the cardiopulmonary exercise test are represented in Table 2. Only 2 children completed a valid peak exercise test in the setting with the FFP2/N95 face mask, whereas 11 were able to achieve this in the normal setting. This was also apparent in the significantly higher peak RER and peak heart rate achieved in the setting without an FFP2/N95 face mask.

**Table 2:** CPET values with and without FFP2 mask as means ± standard deviation assessed with an unpaired *t*-test (* identifies a statistical significance set at *p* < 0.05), as well as the *p*-values for each test and Cohen’s *D* value for effect size

	**No mask**	**FFP2 mask**	***p*-value**	**Cohen’s *D***
RER*	1.01 ± 0.08	0.95 ± 0.08	0.004	0.82
Peak speed (km/h)	10.4 ± 1.5	10.2 ± 1.6	0.508	0.15
$\dot{\mathrm{V}}{\mathrm{O}}_{2}\mathrm{peak}$(ml/min/kg)	42.7 ± 9.5	47.8 ± 12.9	0.097	0.40
Peak HR (bpm)*	191.3 ± 7.4	183.1 ± 18.2	0.016	0.61
$\dot{\mathrm{V}}\mathrm{Epeak}$(ml/min)	49.4 ± 11.9	46.3 ± 14.1	0.551	0.14
O_2_pulse	7.8 ± 1.9	6.9 ± 1.8	0.064	0.45
${\dot{V}}_{E}/{\dot{V}}_{CO2}$slope	33.4 ± 5.9	34.0 ± 5.3	0.689	0.10

*RER* respiratory exchange ratio at the point of maximal exertion, *VO*_*2*_peak oxygen uptake, *Peak HR* peak heart rate, *VEpeak* minute ventilation at peak exercise, *VE/VCO*_*2*_slope correlation between expiratory volume to the volume of CO_2_

There were no significant differences with respect to $\dot{\mathrm{V}}{\mathrm{O}}_{2}\mathrm{peak}$ or peak velocity achieved (vpeak), but there was a tendency to higher values when wearing a face mask.

Peak minute ventilation ($\dot{\mathrm{V}}\mathrm{Epeak}$) was comparable between the two test settings as was the O_2_pulse, a surrogate parameter for cardiac output (s. Table 2). There was also no significant difference in the ${\dot{\mathrm{V}}}_{\mathrm{E}}/{\dot{\mathrm{V}}}_{\mathrm{CO}2}$ slope which is a parameter often used as a marker of ventilatory efficiency, heart failure, and perfusion mismatch.

When asked which of the two test settings had been more strenuous for the children, they could not tell. Some even asked which one should have been more strenuous.

### End-tidal pressures of CO_2_ and O_2_

**Fig. 2:**
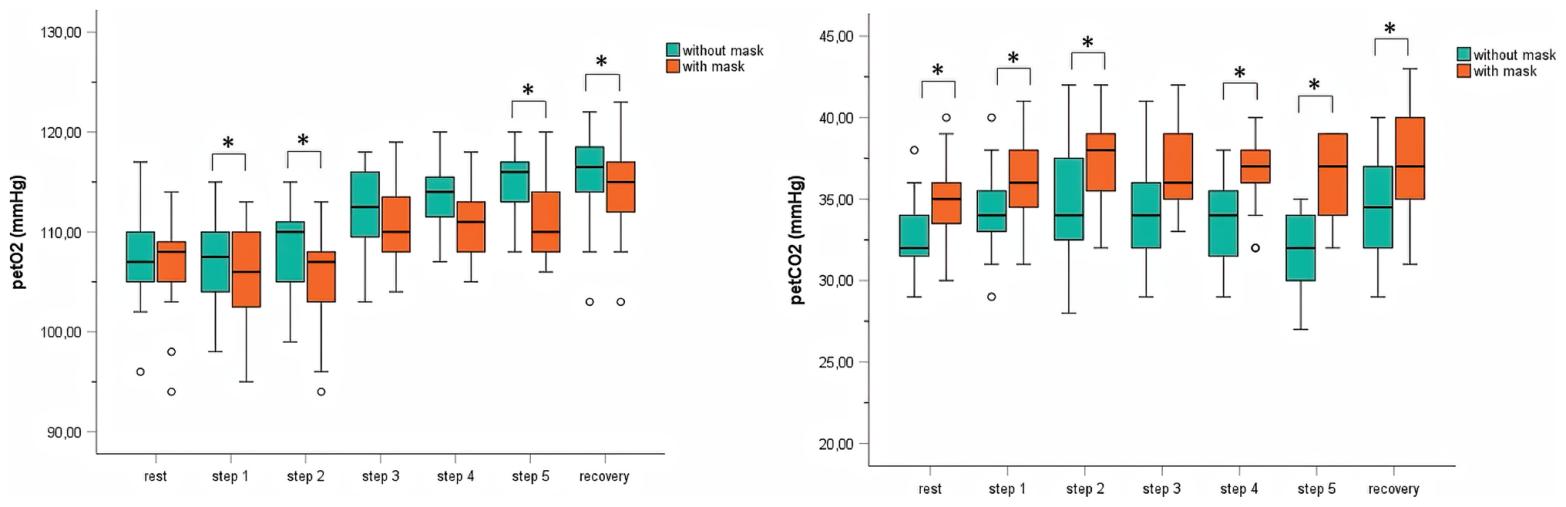
Median, as well as interquartile range as well as minimum and maximum of petO_2_ (mmHg) and petCO_2_ (mmHg) with and without FFP2 mask (* identifies a statistical significance set at *p* < 0.05). Abbreviations: petO_2_, partial pressure of end-tidal oxygen; petCO_2_, partial pressure of end-tidal carbon dioxide

**Fig. 3:**
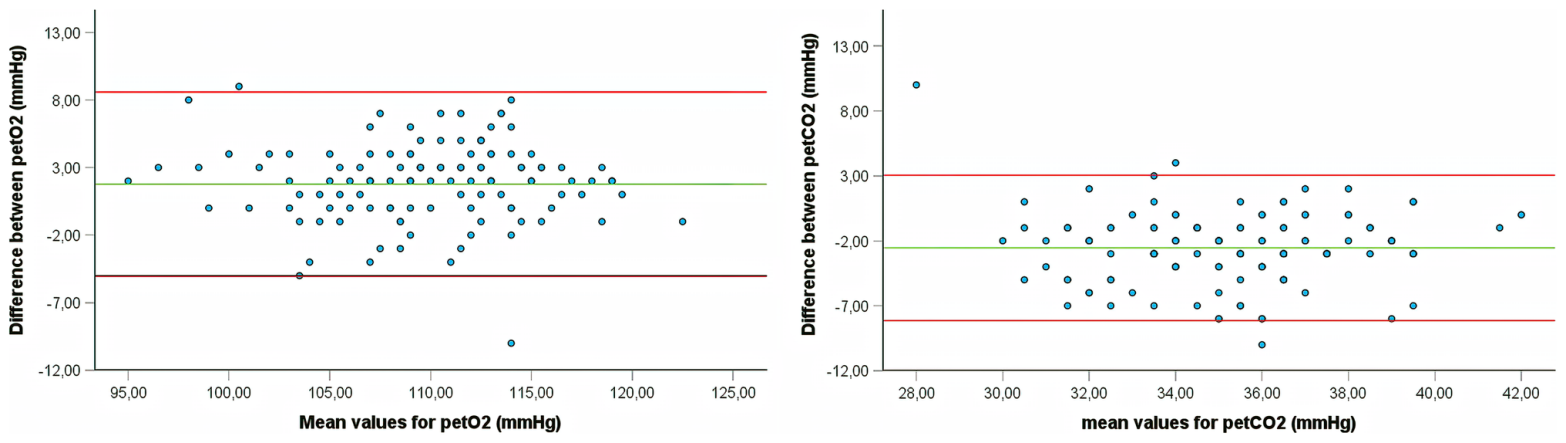
Bland–Altman plot for the petO_2_ (mmHg) and petCO_2_ (mmHg) measurements from all participants. The red lines represent the upper and lower confidence intervals respectively, whereas the green line represents the mean value for the difference between the setting with mask and without mask. Abbreviations: petO_2_, partial pressure of end-tidal oxygen; petCO_2_, partial pressure of end-tidal carbon dioxide

All measurements for end-tidal CO_2_ (pet CO_2_) were significantly higher in the setting with the FFP2/N95 mask reaching significance in nearly all steps except for step 3 (s. Table 3 and Fig. 2). The Bland–Altman plots for the values of pet O_2_ and pet CO_2_ are depicted in Fig. 3. The values for end-tidal O_2_ (pet O_2_) were significantly lower during each step in the mask setting except for the rest situation at the beginning of the test, step 3, and step 4 (s. Table 3 and Fig. 2). All values were well within physiological limits (s. Table 3). Not all children achieved 5 steps on the treadmill.

**Table 3:** petO_2_ and petCO_2_ with and without FFP2 mask as means (mmHg) ± standard deviation assessed with an unpaired *t*-test (* identifies a statistical significance set at *p* < 0.05), as well as *p*-values, Cohen’s *D* value, and Lin’s coefficient for each variable

	**No mask**	**FFP2 mask**	***p*-value**	**Cohen’s *D***	**Lin’s coefficient**
petO_2_ at rest	107.5 ± 4.7	106.5 ± 4.7	0.272	0.260	3.72
petCO_2_ at rest	32.7 ± 2.3	34.9 ± 2.4	< 0.001	0.61	2.09
petO_2_ at step 1	107.0 ± 4.6	105.6 ± 5.0	0.025	0.56	3.53
petCO_2_ at step 1	34.2 ± 2.7	36.3 ± 2.7	0.016	0.61	0.95
petO_2_at step 2	108.1 ± 4.4	105.4 ± 5.1	0.002	0.83	2.78
petCO_2_ at step 2	34.6 ± 3.3	37.3 ± 2.6	< 0.001	1.11	3.48
petO_2_ at step 3	112.0 ± 4.2	110.4 ± 4.4	0.103	0.39	2.12
petCO_2_ at step 3	34.3 ± 3.1	36.8 ± 2.4	0.259	0.27	0.40
petO_2_ at step 4	113.4 ± 3.5	111.8 ± 5.8	0.258	0.29	0.90
petCO_2_ at step 4	33.8 ± 2.8	35.7 ± 4.1	0.048	0.54	2.04
petO_2_ at step 5	114.3 ± 3.7	110.7 ± 3.5	< 0.001	1.79	2.02
petCO_2_ at step 5	32.1 ± 2.6	36.0 ± 2.4	< 0.001	1.79	0.56
petO_2_ at recovery	113.9 ± 4.5	115.4 ± 4.3	0.027	0.55	4.32
petCO_2_ at recovery	36.9 ± 3.3	34.1 ± 3.0	< 0.001	0.94	1.24

*petO*_*2*_ partial pressure of end-tidal oxygen, *petCO*_*2*_ partial pressure of end-tidal carbon dioxide

Not all children achieved all the steps during the treadmill test. All children were able to perform up to step 3, then one dropped out, and then a further 6 (7 in total) could not finish the last step.

## Discussion

This is the first study investigating the effects of wearing an FFP2/N95 mask during an incremental treadmill test using CPET in children.

Interestingly, the children in this study could not tell, which test had been more strenuous and many even asked which one should have been more strenuous. This is in concordance with previous studies in children, in which cloth face masks did not affect ratings of perceived exertion during a progressive square-wave test [36] or during a submaximal sit-to-stand test [37]. So far, the only studies investigating the impact of wearing FFP2/N95 face masks on exercise tolerance have been limited to adults with higher degrees of rating of perceived exertion, dyspnea, fatigue, and thermal sensation [17, 27–30, 33, 46].

Still, children ended the treadmill test at a significantly lower RER when being fitted with the additional FFP2 mask. The respiratory exchange ratio (RER) allows for an estimation of the actual exertion of the subject as it represents the ratio of exhaled CO_2_ over inhaled O_2_. With the accumulation of lactate, more CO_2_ needs to be exhaled pushing the ratio over 1. When the FFP2/N95 mask was fitted over the CPET mask, the mean RER was below 1, suggesting insufficient exertion during these tests. The reason for this observation is unclear, since the children did not observe any discomfort, but apparently could not reach peak exertion when fitted with the additional mask. However, the phenomenon that face masks can hinder sufficient exertion, with lower metabolic responses when wearing a face mask during resistance exercise, has been observed previously [17, 19, 47]. One possible explanation provided states that low air supply when wearing a mask could influence the central nervous system which then stops the exercise in order to prevent biological damage [19]. So far, no deleterious effects of wearing a face mask on biological systems have been observed [21]. Another explanation states that wearing a face mask is perceived as subjectively disturbing leading to an increased perception of exertion and in consequence a negative impact on exercise tolerance [17].

Another parameter pointing towards a lower peak exertion is the heart rate at $\dot{V}{O}_{2}peak$ which was also significantly lower in the test with the additional FFP2/N95 mask. Wearing cloth face masks during a progressive square-wave test did not show any differences in peak HR in children [36]. Nor could any significant differences be discerned when studying the impact of FFP2/N95 masks on the peak heart rate in adults [27, 31, 33, 46]. A decreased heart rate when wearing an FFP2/N95 mask was only observed in one other study in patients with heart failure [30]. However, the reasoning that a reduced ability of the failing heart to adapt leads to this difference cannot be applied to the children studied here, as they were all healthy. Most likely, the previously mentioned increased perception of exertion [17], even if not verbally acknowledged by the children, led to a premature test ending.

Interestingly, the fact that the peak RER and peak HR differed significantly between the two test settings could not be observed in any other CPET parameter established during the two tests. In terms of performance, the children achieved comparable top speeds with and without the FFP2/N95 face mask. The data in the adult population is controversial with regard to this parameter as some meta-analyses also observed no difference in peak power output [31] while others did [46]. Since the data is not unanimous, we believe that peak exertion should not be expected in children when wearing FFP2/N95 masks and the grading of performance in PE classes should therefore be limited in our opinion.

Since no studies have used CPET to objectify the effects of mask wearing in children, only studies in the adult population can be used for comparison of these parameters. Wearing FFP2/N95 masks led to a significant decrease in $\dot{V}{O}_{2}peak$ not observed when wearing surgical masks [29, 31, 46]. This was also true in well-trained athletes [33] and patients with heart failure [30]. This change is explained by an increase in airway resistance induced by a reduction in alveolar ventilation when wearing a mask [46]. On top of the increased airway resistance, the multiple layers and materials included in the construction of the FFP2/N95 mask increase the inspiratory resistance, thus decreasing the amount of oxygen inhaled, which results in a reduction in $\dot{V}{O}_{2}$ [48]. Even though the differences between the two test settings did not reach significance with regard to $\dot{V}{O}_{2}peak$ in our study, the values achieved by the children were actually higher when wearing the mask. Bearing in mind that they achieved significantly lower values for peak exertion, this suggests that they may have achieved higher values for $\dot{V}{O}_{2}peak$. One possible explanation for this difference could be the fact that we applied the FFP2/N95 mask on the exterior of the CPET mask in order to measure the true variables as in- and exhaled by the children. The other studies fitted the mask on the inside, which may have lowered the actual measurement. On the other hand, the fact that the peak power output was comparable between the two test settings suggests that the children had a higher oxygen consumption when wearing an FFP2/N95 face mask, so wearing the mask may be more strenuous after all. In other words, when wearing an FFP2/N95 face mask, the oxygen consumption is higher for the same workload which suggests more strain for the same workload.

Most often, a decrease in pulmonary function is observed when wearing FFP2/N95 face masks, including a reduction in $\dot{V}E$ and $\dot{V}E/VC{O}_{2}$ [29, 31, 46]. The observed reductions are believed to be caused by increased inspiratory resistance [46]. But, as the level of change for $\dot{V}E/VC{O}_{2}$ was limited and remained within normal range and the reduction in $\dot{V}E$ was relatively large, gas leakage from the CPET mask as a consequence of insufficient seal caused by wearing a face mask underneath could also be the cause [46]. In our study, both parameters were comparable between the two test settings. Either children don’t suffer from the increase in inspiratory resistance caused by the FFP2/N95 mask or the fact that the mask was applied on the outside of the CPET mask ensured sufficient seal to measure true values. The comparable measurements of $\dot{V}{O}_{2}peak$ and $\dot{V}Epeak$ at lower objective exertion (lower RER and lower peak HR) suggest that the children may have achieved higher peak values if they had kept going. One possible explanation for this paradoxical result is the measurements for petCO_2_ which were significantly higher during each step. Arterial carbon dioxide levels control breathing and thus oxygen intake. Possibly the higher values for petCO_2_ caused the participants to breathe harder and thus increase their oxygen consumption. However, since peak exertion was not achieved by all participants, this cannot be verified with the current data.

Another benefit of applying the FFP2/N95 mask on the outside of the CPET mask was that we were able to measure end-tidal values of CO_2_ and O_2_ on the inside of the FFP2/N95 mask as in real life. In many countries, PE lessons and PA in sports clubs were limited during the pandemic because it was feared that wearing face masks might impair oxygen uptake and cause carbon dioxide retention [35, 49]. Accordingly, higher values for PetCO_2_ when wearing an FFP2/N95 have been recorded during graded exercise testing due to CO_2_ rebreathing [46]. We observed the same phenomenon in children wearing FFP2/N95 face masks with significantly higher PetCO_2_ values in almost every step of the graded treadmill test, even at rest and during recovery. However, since the values remained well below the upper limit of normal, true carbon dioxide retention was not observed. Neither did the values for PetO_2_ reveal dangerously low levels, but the values were significantly lower when wearing the additional mask than in the normal setting, as observed previously [29, 46].

Wearing an FFP2/N95 face mask during physical activity in school or in sports clubs therefore seems to have no major negative impact on physiological demands of physical activity of any intensity in children. On the contrary, children seem to be able to adapt to the intensity of their exercise without being aware of a potential higher energy demand. However, discontinuing PE lessons or PA in sports clubs due to the potential physiological risks from wearing face masks can have serious long-term effects on the physical, psychological, and social well-being of children [2, 9–11, 34]. This is especially true if PE in school is one of the only sources of PA for some children. It is debatable whether pushing children to perform at their best is recommendable in times of mandatory face mask wearing, but performing sports should remain part of the regular schedule for all children at all times.

### Limitations

Even though the number of participants in this study is rather low, it yielded significant differences between the two test settings. This is especially true for the main parameters: end-tidal O_2_ and end-tidal CO_2_. This study was mainly conducted to investigate the effect of wearing an FFP2 face mask on the gas exchange during physical activity. The Bland–Altman plots as well as the Lin coefficients were able to show that the cohort was large enough for being able to show a significant influence of the mask on the end-tidal gas values (s. Fig. 3). A larger cohort may have provided significant results for the remaining parameters such as peak oxygen uptake, peak heart rate, or peak ventilation but this is debatable since the values for gas exchange did not reach pathological values and the children did not experience any difference with respect to peak exertion between the two test settings. Furthermore, the number of investigated participants is comparable to previous studies in children and adults, investigating similar research questions.

Since the study was conducted in children, values for oxygen saturation were not included. The oxygen measurements in children using regular CPET equipment often yield unreliable results. We, therefore, preferred not to use these measurements and preferred to rely on objectifiable values like petO_2_.

We attached the FFP2/N95 face mask on the outside of the CPET mask in order to sample all values on the inside of the mask as experienced by the subject. This method differs from previous studies and a comparison with other studies is thus hampered. Still, we believe this to be the more precise and realistic method to measure true oxygen, carbon dioxide, and breathing parameters. This setup also made blinding impossible, as the children were able to see whether the test was being carried out with or without a mask. However, since none of them were able to tell which of the test situations was more strenuous, the bias of knowing which setup contained the mask may not have been so strong as in adults who might be prejudiced about the expected outcome of running with a mask.

A rating of perceived exertion (RPE) was not included in this study because the children were younger than 10 years of age. Children these young are less reliable with regard to RPE [50]. However, when investigating the effects of wearing a face mask in older children, such a scale should be included to estimate subjective exertion.

## Conclusion

In this study, no significant differences in the cardiorespiratory function at peak exercise could be discerned when wearing an FFP2/N95 face mask. Even though the end-tidal values for CO_2_ were significantly higher and for end-tidal O_2_ significantly lower during the whole exercise test, these values did not reach pathological values. Furthermore, the children stopped the exercise at a lower RER and HR, even though they could not tell which setup was more strenuous. This finding suggests that children might be subconsciously aware of the higher strain and stop exercising early. Since the deleterious effects of limiting PA due to the mandatory wearing of face masks are well known, the minor physiological effects these masks seem to have, stopping PE lessons as a consequence, seems premature and should be reconsidered in the future.

## Data Availability

Data can be made available upon request.

## Abbreviations

ANS: Autonomic nervous system
BPD: Bronchopulmonary dysplasia
BR: Breathing rate
CPAP: Continuous positive airway pressure
CPET: Cardiopulmonary exercise testing
CRF: Cardiorespiratory fitness
HR: Heart rate
HRR: Heart rate recovery
OUES: Oxygen uptake efficiency slope
PA: Physical activity
Peak HR: Peak heart rate
peakRER: Respiratory exchange ratio at maximal exertion
PE lessons: Physical education lessons
petCO_2_: End-expiratory gas exchange for carbon dioxide
petO_2_: End-expiratory gas exchange for oxygen
RER: Respiratory exchange ratio
RSV: Respiratory syncytial virus
SpO_s_: Oxygen saturation
$\dot{\mathrm{V}}C{O}_{2}$: Carbon dioxide elimination
$$\dot{V}E$$: Minute ventilation
$\dot{V}Epeak$: Peak minute ventilation
$$\dot{V}E/\dot{V}C{O}_{2}$$: Breathing efficiency
$$\dot{V}{O}_{2}$$: Oxygen uptake
$\dot{V}{O}_{2}peak$: Peak oxygen uptake
VT_1_: Ventilatory threshold 1
